# Sleep and Circadian Rhythms in Survivors of Acute Respiratory Failure

**DOI:** 10.3389/fneur.2020.00094

**Published:** 2020-02-14

**Authors:** Pei-Lin Yang, Teresa M. Ward, Robert L. Burr, Vishesh K. Kapur, Susan M. McCurry, Michael V. Vitiello, Catherine L. Hough, Elizabeth C. Parsons

**Affiliations:** ^1^School of Nursing, University of Washington, Seattle, WA, United States; ^2^Division of Pulmonary, Critical Care and Sleep Medicine, University of Washington, Seattle, WA, United States; ^3^Department of Psychiatry and Behavioral Sciences, University of Washington, Seattle, WA, United States; ^4^VA Puget Sound Health Care System, Seattle, WA, United States

**Keywords:** acute respiratory distress syndrome, critical illness, sleep, circadian rhythm, actigraphy

## Abstract

**Background:** Little is known about sleep and circadian rhythms in survivors of acute respiratory failure (ARF) after hospital discharge.

**Objectives:** To examine sleep and rest-activity circadian rhythms in ARF survivors 3 months after hospital discharge, and to compare them with a community-dwelling population.

**Methods:** Sleep diary, actigraphy data, and insomnia symptoms were collected in a pilot study of 14 ARF survivors. Rest-activity circadian rhythms were assessed with wrist actigraphy and sleep diary for 9 days, and were analyzed by cosinor and non-parametric circadian rhythm analysis.

**Results:** All participants had remarkable actigraphic sleep fragmentation, 71.5% had subclinical or clinical insomnia symptoms. Compared to community-dwelling adults, this cohort had less stable rest-activity circadian rhythms (*p* < 0.001), and weaker circadian strength (*p* < 0.001).

**Conclusion:** Insomnia and circadian disruption were common in ARF survivors. Sleep improvement and circadian rhythm regularity may be a promising approach to improve quality of life and daytime function after ARF.

## Introduction

Survivors of Acute Respiratory Distress Syndrome (ARDS) experience impairments in physical, psychological, and cognitive functioning, as well as quality of life (1–4). Sleep disorders may play an important role in post-intensive care unit (ICU) syndrome, defined as a constellation of cognitive, psychological and physical impairment lasting months to years after discharge (5). A systematic literature review found that sleep disturbances affected an estimated 67% of ARDS survivors at 1-month post-hospitalization and 39% beyond 1-month post-hospitalization (6). The majority of these studies use self-report measures that focus on sleepiness or insomnia (6, 7). Few studies have incorporated objective sleep measures that could identify circadian rhythm (also known as sleep-wake) disorders for which ARDS survivors are at risk (7).

Many biobehavioral processes in humans, including sleep-wake behavior, follow a diurnal pattern, often called a circadian rhythm. Circadian rhythms are endogenous rhythmic patterns in multiple physiological and behavioral processes such as sleep-wake patterns, rest-activity patterns, mood, cognitive function, body temperature, heart rate, and hormone secretions that are entrained to a 24-h day to enable individuals to anticipate and adapt to periodic environmental changes (8–10). Optimal circadian entrainment, exhibiting a 24-h period with an appropriate amplitude (strength) and phase (timing), is essential for the optimization of physical, psychological, and behavioral functions and overall human health (9, 10). Abnormalities in the circadian rhythm have been identified in the acute ICU period, including a dampened circadian amplitude, and/or a delayed phase of physiological circadian biomarkers (i.e., melatonin, cortisol, cytokines) (11–13). Left untreated, circadian disruption may contribute to persistent sleep complaints including insomnia (9, 10) and/or influence mood and cognition (14, 15). However, to date, it is not known whether circadian rhythms are disrupted after hospital discharge among ARDS survivors.

Wrist actigraphy is a non-invasive, objective measure of sleep-wake patterns and circadian rest-activity rhythms recordable for extended periods of time in a natural environment. Estimates of sleep parameters (i.e., sleep onset and offset, total sleep time, sleep efficiency) and rest-activity circadian rhythms with actigraphy in conjunction with sleep diaries have been validated by polysomnography (PSG) and other circadian biomarkers such as melatonin and core body temperature (16–20). Wrist actigraphy has been accepted as a reliable surrogate measure of sleep and entrained endogenous circadian rhythms in both clinical and research settings (16–18, 21). To our knowledge, there are no published studies using actigraphy in conjunction with sleep diary to identify circadian disruption among ARDS survivors.

The purpose of this pilot study was to: (1) examine sleep duration, sleep quality, and rest-activity circadian rhythms as measured by sleep diary and actigraphy in ARDS survivors 3 months after hospital discharge; and (2) compare sleep and circadian rest-activity rhythms of ARDS survivors to community dwelling adults in the U.S. A better understanding of sleep and circadian rest-activity rhythms in ARDS survivors after hospital discharge may provide new knowledge for the development of interventions to improve sleep and daytime function on the ARDS population.

## Materials and Methods

### Participants and Procedures

This study is a subset analysis from a prospective observational cohort pilot study named **S**leep **a**nd **R**ecovery after **A**cute Respiratory Failure (**SARA study**). SARA recruited 21 patients with acute respiratory failure admitted to an urban medical center in the Pacific Northwest, a tertiary care hospital from December 2012 to August 2013. Patients in the medical and surgical ICU were screened consecutively for eligibility in this prospective observational cohort study. Eligible patients (or their healthcare proxies) were approached before or shortly after hospital discharge (within 1 week of discharge) and invited to participate in the study. Enrolled patients included both medical and surgical ICU patients with a variety of primary diagnoses and varying lengths of stay. Inclusion criteria included (1) >18 years, (2) admitted to the ICU, and (3) required ≥48 h of mechanical ventilation. Participants were excluded if they had (1) chronic mechanical ventilation, (2) primary neurological injury or diagnosis, or pregnancy, or (3) inability to obtain patient or surrogate consent. The SARA study was reviewed and and approved by the University Human Subjects Institutional Review Board (No. 43516). Informed consent was obtained from all participants prior to data collection. Patients enrolled in the study were evaluated prospectively using sleep questionnaires, actigraphy and sleep diary. At 3 months post-discharge, study evaluation included a survey of sleep and quality of life symptoms, and 9 days of actigraphy and sleep diary. Of 21 patients enrolled in the SARA study, this subset analysis included the 14 patients with sleep diary and actigraphy data (the participant rate: 66.7%, two patients were lost to follow-up, 1 withdrew from the study, 4 refused actigraphy).

### Measures

#### Sleep Characteristics

Sleep characteristics were evaluated objectively by actigraphy and subjectively by sleep diary for 9 consecutive days at home.

##### Objective sleep estimates

Participants were asked to wear a wrist actigraphy (Actiwatch2, Phillips-Respironics) on the non-dominant wrist to record raw activity counts in 1 min epochs. Actigraphy data were scored as sleep or wake with medium (40 activity count threshold) wake sensitivity settings under Actiware software (Mini-Mitter Philips Respironics, Inc.) (6, 19, 20, 22). The onset and offset of the rest intervals were determined by the sleep diaries and was based on the American Academy of Sleep Medicine (AASM) scoring actigraphy standard criteria (19, 20, 23). Five actigraphy sleep parameters were included in this study: (1) sleep onset, defined as the time of the first period of 10 or more consecutive minutes of immobility; (2) sleep offset, defined as the time of the last period of 10 or more consecutive minutes of immobility; (3) total sleep time (TST_A_), defined as the total amount of nocturnal sleep duration in hours during the nighttime rest period scored as sleep; (4) actigraphy sleep efficiency (SE_A_), defined as the ratio of TST to the nighttime rest period (multiplied by 100 to yield a percentage); and (5) amount of wake, defined as the wake time after sleep onset (WASO) in minutes.

##### Subjective sleep estimates

Three sleep parameters derived from sleep diary included (1) total sleep time (TST_D_), defined as the time participants reported in the item as “the total amount of time they slept at night;” (2) diary-derived SE (SE_D_), defined as the ratio of TST_D_ to total time in bed (multiplied by 100 to yield a percentage); and (3) self-reported sleep quality, assessed by how participant rated the item “when I woke up for the day, I felt” on the level of refreshed, somewhat refreshed, or fatigued.

##### Insomnia symptoms

Participants were administered the Insomnia Severity Index (ISI) to measure their perception of insomnia symptoms over the past 2 weeks. The ISI is a self-reported seven-item questionnaire designed to assess the severity of sleep problems (e.g., sleep-onset, difficulties in maintenance), satisfaction with current sleep patterns, interference with daily functioning, the impairment of quality of life and distress about sleep problems (24). Each item is rated with a five-point Likert scale (“not at all” to “extremely”) and total score ranges from 0 to 28 with higher scores indicating greater severity of insomnia symptom. An ISI value between 8 and 14 was used to identify subclinical insomnia; a cut-off value of ≥15 was used to identify clinically significant insomnia (24). The ISI has established reliability and validity for survivors of critical illness (25, 26).

#### Rest-Activity Circadian Rhythm

**Figure 1:**
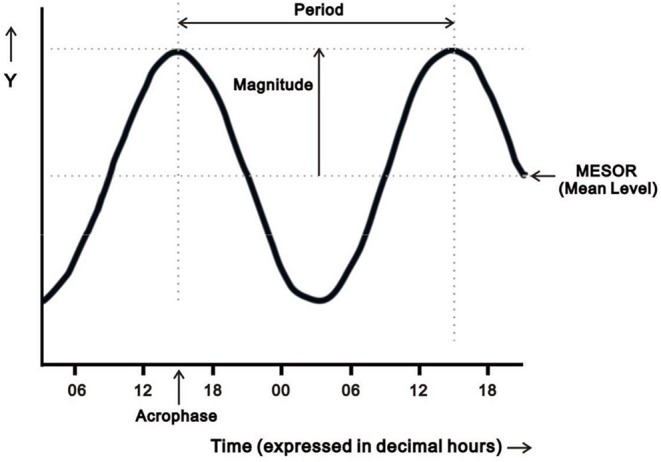
Diagram illustrating four parameters of the oscillation from cosinor analysis: mesor (the estimated mean activity count of the fitted 24-h pattern), period (the duration of a full cycle), magnitude (mesor-to-peak difference, indicating robustness/strength of rhythm), and acrophase (the time of peak activity). Adapted from Cornelissen (28).

Rest-activity circadian rhythms derived from actigraphy recordings in this study were analyzed by two different methods to account for both normal and non-normal distributions of data, using traditional parametric cosinor analysis and non-parametric circadian rhythm analysis (NPCRA) (16, 27, 28). Analysis of circadian rhythms requires specialized statistical analyses to account for the periodicity of the data, the distribution of the data, and temporal changes over time. Cosinor analysis is a traditional approach to characterize rest-activity circadian rhythms, in which a cosinor curve with a period of 24 h is fit to a multiple-day time series of measurements of raw activity counts by using least-squares methods (27–31). Three cosinor parameters were included in this study: (1) mesor, defined as mean activity count of the fitted 24-h circadian rhythm pattern; (2) magnitude, defined as mesor-to-peak difference, indicating half estimated extent of the variation within the cycle; and (3) acrophase, defined as highest activity timing in the cycle expressed in decimal hours [Figure 1; (28, 29)]. These three parameters were used to characterize the circadian phase (timing) and circadian strength. Clinically, acrophase is the measure of circadian phase; mesor and magnitude reflect the robustness of rest-activity circadian rhythms.

As the assumed temporal distribution of cosinor analysis may not always fit the real-world rest-activity patterns well, we also performed a non-parametric analysis (NPCRA) to make our data analysis more robust (32). Six NPCRA variables were included in this study: (1) interdaily stability (IS) provides an estimate of stability of rest-activity rhythms across days, ranging from 0 to 1, and a higher IS value indicates a better synchronization to 24-h environmental cues; (2) intradaily variability (IV) provides the estimate of fragmentation of the 24-h rest-activity rhythms, ranging from 0 to 2, and a higher IV value is found in individuals who have worse sleep efficiency or greater fragmented circadian rhythm patterns; (3) M10 midpoint is determined by the midpoint of the 10 most active consecutive hours, indicating the time of highest activity; (4) L5 midpoint is determined by the midpoint of the 5 least active consecutive hours, indicating the time of lowest activity; (5) amplitude is determined by difference between M10 and L5 levels, indicating the difference in activity level of highest and lowest activity periods; and (6) relative amplitude (RA) is determined by the amplitude divided by the sum of L5 and M10 levels, ranging from 0 to 1, and a higher RA indicates stronger circadian rhythm strength (27, 29, 33).

#### Demographic and Clinical Characteristics

Trained research personnel extracted demographic (i.e., age and gender) and clinical characteristics including pre-existing comorbidities (heart disease, sleep apnea, hypertension, diabetes, and depression), body mass index (BMI), diagnosis at ICU admission (trauma or medical), length of hospital stay and ICU stay, duration of medical ventilation, PaO_2_/FiO_2_ ratio, and illness severity at ICU admission based on Acute Physiology and Chronic Health Evaluation II classification system (APACHE II) (34) from electronic medical record. Participants were asked about living situation and employment status as well as if they needed any assistance with any of eight instrumental activities of daily living (IADLs) including using the telephone, walking, shopping for groceries, preparing meals, doing housework or handyman work, doing laundry, taking medication, and managing money (35) on a scale of 0 (unable to do at all), 1 (need some help), and 2 (need no help). The summed individual IADL scores were used to create a global IADL score, indicative of patient functional status; global IADL scores could range from 0 (low function, dependent) to 16 (high function, independent).

### Statistical Analysis

Descriptive statistics were used to describe the demographic and clinical characteristics of the participants. As only actigraphy sleep data with matching sleep diary data were included in the analyses, participants had between 6 and 9 days of concurrent actigraphy sleep data (median = 9 days, five participants had <9 days of concurrent data). Actigraphy-measured sleep onset and offset, TST_A_, SE_A_, and WASO plus self-reported TST_D_ and SE_D_ from the sleep diary were averaged over consecutive study days for each participant. The sleep characteristics were skewed; consequently, medians and interquartile ranges (IQR) were reported for the actigraphy and self-report sleep measures.

Actigraphy circadian outcomes were analyzed by cosinor analysis and NPCRA analysis to quantify 24-h rest-activity circadian rhythms for each participant. Actigraphy raw activity data were transformed using natural log (ln) function followed by fitting a 24-h cosine curve through regression analysis. Given the activity count data were highly skewed, natural log transformation was able to make the activity count data at each time point more uniform and balanced over a 24-h period, especially important for the low-activity nighttime period (36). Circadian outcomes were presented as means ± standard deviation (SD) to depict rest-activity circadian rhythms.

Given the nature of this pilot study with the lack of comparison group, each of the mean actigraphy circadian parameters, TST_A_, and SE_A_ used Hedges' G effect sizes to compare with those from a sample of 578 community-dwelling U.S. adults, which have been described in detail elsewhere (37). Data analyses were performed using SPSS (v 25).

## Results

### Participant Demographic and Clinical Characteristics

Table 1 shows the demographic and clinical characteristics of the study sample. The mean age of the sample was 49.1 ± 15.9 years; 71% were male and lived independently. Fifty-seven percent were trauma patients and 79% met the criteria for moderate and severe ARDS (PaO2/FiO2 ratio ≤ 200) (38). The mean premorbid IADL score was 12.8 ± 2.5, indicating mild functional limitations, yet only 14% (*n* = 2) were full-time employed.

**Table 1:** Demographic and clinical characteristics of ARDS survivors (*n* = 14).

**Variables^a^**	**Total sample**
Demographics	
Age, years	49.1 ± 15.9
Male, *n* (%)	10 (71)
Body mass index, kg/m^2^	27.4 ± 3.5
Living situation	
House/apartment (independent)	10 (71)
Rehabilitation facility	2 (14)
Others	2 (14)
IADL scores (0-16)	12.8 ± 2.5
Full time employment, *n* (%)	2 (14)
Pre-existing comorbidities, *n* (%)	
Heart disease	5 (36)
Sleep apnea	2 (14)
Hypertension	5 (36)
Diabetes	4 (29)
Depression	1 (7)
ICU characteristics	
Diagnosis at ICU admission, *n* (%)	
Trauma	8 (57)
Medical	6 (43)
Disease severity, APACHE II score	24.5 ± 4.9
PaO_2_/FiO_2_ ratio	163.8 ± 90.0
PaO_2_/FiO_2_ ratio ≤ 200, *n* (%)	11(79)
Length of hospital stay, days	21 ± 17.5
Length of ICU stay, days	11 ± 8.8
Duration of mechanical ventilation, hours	175.8 ± 135.0

*ARDS, acute respiratory distress syndrome; IADL, instrumental activities of daily living; ICU, intensive care unit; APACHE II, acute physiology and chronic health evaluation II (APACHE II) classification system*.

a *Reported as mean ± standard deviation unless specified*.

### Sleep Characteristics in ARDS Survivors

Table 2 summarizes the actigraphy and sleep diary data and insomnia symptom scores. The median TST_A_ was 7.8 h (IQR: 6.2–8.3 h), the median WASO was 59.8 min (IQR: 45.0–92.8 min) and the median SE_A_ was 81.9% (IQR: 78.9–86.0%). Based on sleep diary data, the median TST_D_ was 6.6 h (IQR: 5.3–6.9 h), with a median SE_D_ of 70.0% (IQR: 62.2–80.6%). The median ISS score was 12.0 (IQR: 6.8–15.0). 42.9% of participants had subclinical insomnia (8 ≤ ISS score ≤ 14), and 28.6% had clinically significant insomnia (ISI score ≥15).

**Table 2:** Sleep characteristics measured by actigraphy, sleep diary, and symptom survey (*n* = 14).

**Variables**	**Median (IQR)**
**Actigraphy**	
Sleep onset^a^	23:29 (22:11–00:30)
Sleep offset^a^	7:24 (6:11–09:19)
TST_A_, hours	7.8 (6.2–8.3)
TST_A_ <7 h, *n* (%)	5 (35)
SE_A_, %	81.9 (78.9–86.0)
SE_A_ <85%, *n* (%)	9 (64.3)
WASO, minutes	59.8 (45.0–92.8)
WASO >30 min, *n* (%)	14 (100)
**Sleep diary**	
TST_D_, hours	6.6 (5.3–6.9)
TST_D_ <7 h, *n* (%)	11 (79)
SE_D_, %	70.0 (62.2–80.6)
SE_D_ <85%, *n* (%)	11 (79)
Self-reported sleep quality^b^	
Refreshing sleep >3 nights, *n* (%)	2 (14.3)
Somewhat refreshing night >3 nights, *n* (%)	9 (64.3)
Fatigued sleep >3 nights, *n* (%)	4 (28.6)
**Insomnia symptoms, ISI score**	12.0 (6.8–15.0)
Clinical insomnia (ISI ≥15), *n* (%)	4 (28.6)
Subthreshold (8 ≤ ISI ≤ 14), *n* (%)	6 (42.9)
No clinically significant insomnia (ISS <8), *n* (%)	4 (28.6)

*IQR, interquartile range; TST_A_, total sleep time estimated by actigraphy; SE_A_, sleep efficiency estimated by actigraphy; WASO, wake after sleep onset; TST_D_, total sleep time estimated by sleep diary; SE_D_, sleep efficiency estimated by sleep diary; ISS, insomnia severity index*.

a *Sleep onset, sleep offset times are presented in 24-h clock format*.

b *Due to missing data (18 number of days), 96 number of days were included to examine self-reported sleep quality*.

### Rest-Activity Circadian Rhythm

Table 3 summarizes circadian rest-activity measures, as well as TST_A_ and SE_A_ and compares them with a community-dwelling sample of US adults (37). Additionally, comparisons of age and BMI between the two samples were performed to examine the potential effects of age and BMI on rest-activity circadian rhythms (37, 39). No significant differences in age and BMI between two samples were found, indicating the sample of community-dwelling US adults was comparable to our sample on these important rest-activity measures.

**Table 3:** Comparisons of the results from actigraphy rest-activity circadian rhythm and sleep data with those from a sample of community-dwelling US adults^a^.

**Parameters**	**ARDS survivors (*n* = 14)**	**Community-dwelling US adults (*n* = 578)**	**E.S_**.(G)**_**	***p***
Age, years	49.1 (15.9)	51.9 (14.9)	−0.19	0.489
Body mass index, kg/m^2^	27.4 (3.5)	27.5 (6.0)	−0.02	0.951
**24-h rhythm stability /variability**				
*NPCRA* IS	0.32 (0.10)	0.49 (0.12)	−1.42	**<0.001**
*NPCRA* IV	0.55 (0.18)	0.86 (0.24)	−1.30	**<0.001**
**Activity strength**				
*Cosinor* Mesor, ln counts	2.9 (0.43)	3.9 (0.5)	−2.01	**<0.001**
**24-h rhythm strength**				
*Cosinor* Magnitude, ln counts	1.8 (0.39)	2.6 (0.5)	−1.61	**<0.001**
*NPCRA* Amplitude, ln counts	3.23 (0.68)	7.0 (0.3)	−12.03	**<0.001**
*NPCRA* Relative Amplitude (RA)	0.64 (0.11)	0.77 (0.11)	−1.18	**<0.001**
**24-h rhythm phase**				
*Cosinor* Acrophase, decimal hours	15.40 (2.05)	14.6 (1.3)	0.61	0.261
*NPCRA* L5 midpoint, decimal hours	3.44 (2.49)	2.7 (1.3)	0.55	0.288
*NPCRA* M10 midpoint, decimal hours	15.26 (2.64)	14.2 (1.6)	0.65	0.158
**Actigraphy sleep parameters**				
Total sleep time, hours	7.4 (1.2)	6.90 (0.97)	0.51	0.146
Sleep efficiency, %	81.7 (4.8)	85.6 (7.10)	−0.55	**0.010**

*ARDS, acute respiratory distress syndrome; NPCRA, non-parametric circadian rhythm analysis; Ln, natural log; IS, interdaily stability; IV, intradaily variability; E.S_.(G)_, Hedges' G effect sizes. Bold values denote the significance at the p < 0.05 level*.

a *Data reported by Cespedes et al. (37)*.

#### 24-h Rhythm Stability/Variability

ARDS survivors had a mean IS of 0.32 (±0.10) compared to a typical value of 0.6 in healthy adults (27), suggesting ARDS survivors had a less stable rest-activity rhythm across the days. ARDS survivors had a mean IV of 0.55 (±0.18) which was congruent to the typical value of <1 for healthy adults (27). This suggests that ARDS survivors have a fairly regular rest-activity rhythm within a single day. ARDS survivors had significantly less stable rest-activity circadian rhythms across days [Hedges' G effect size, E.S._(G)_ for IS = −1.42, *p* < 0.001], and less fragmented rest-activity circadian rhythms within a single day [E.S._(G)_ for IV = −1.30, *p* < 0.001] compared with the sample of community-dwelling US adults. Less stability of rest-activity circadian rhythms across days in ARDS survivors could represent the suboptimal entrainment of endogenous circadian rhythms to environmental and social cues (37).

#### 24-h Rhythm Phase

ARDS survivors exhibited later timing for peak activity [E.S._(G)_ for acrophase = 0.61, E.S._(G)_ for M10 midpoint = 0.65] and rest periods [E.S._(G)_ for L5 midpoint = 0.55], and it might indicate a delayed circadian phase (also known as circadian phenotype) in ARDS survivors (40); however, the mean acrophase, L5, and M10 midpoint values were not significantly different in the two samples. ARDS survivors exhibited greater variances in peak activity (SD for acrophase = 2.05 vs. 1.3, SD for M10 midpoint = 2.64 vs. 1.6, respectively) and rest periods (SD for L5 midpoint = 2.49 vs. 1.3, respectively) compared with the sample of community-dwelling US adults, indicating higher heterogeneity of circadian phase in ARDS survivors.

#### Activity Strength and 24-h Rhythm Strength

A large effect size in mesor [E.S._(G)_ = −2.01, *p* < 0.001], magnitude [E.S._(G)_ = −1.61, *p* < 0.001], amplitude [E.S._(G)_ = −12.03, *p* < 0.001], and RA [E.S._(G)_ = −1.18, *p* < 0.001] were found in ARDS survivors of the sample compared with the sample of community-dwelling US adults. These results indicate that ARDS survivors were less active and had weaker rest-activity circadian rhythms compared to community-dwelling adults.

#### Sleep Quantity and Efficiency

Both TST_A_ [E.S._(G)_ = 0.51, *p* = 0.15] and SE_A_ [E.S._(G)_ = −0.55, *p* = 0.01] produced moderate effect sizes, but only SE_A_ was statistically *significant*, indicating that ARDS survivors had a longer objective total nighttime sleep time but lower sleep efficiency at 3 months after hospital discharge relative to the comparison sample.

## Discussion

This study provides preliminary data regarding rest-activity circadian rhythms and sleep quality after discharge among acute respiratory failure survivors. At 3 months post-discharge, our cohort of ARDS survivors exhibited subclinical and clinical insomnia symptoms, supported by findings from actigraphy (sleep fragmentation) and sleep diaries (reduced sleep time and quality). Survivors' rest-activity circadian rhythms displayed less stability and lower amplitude compared to that of a published community-dwelling population. To the best of our knowledge, this is the first study to identify persistent circadian rhythm disruption in ARDS survivors after hospital discharge.

### Sleep Disruption in ARDS Survivors

Resolving the literature on sleep disruption after critical illness can be challenging due to the heterogeneity of ICU populations across studies. ICU survivors are a heterogenous group of patients with a variety of diagnoses, pre-existing comorbidities, severity of illness, and long-term outcomes. ARDS survivors are a useful population to examine the link between sleep and/or disruption and functional impairment, because this population shares a common pathophysiology (respiratory failure) and has a high prevalence of long-term functional impairment (4). Our goal in this study was to identify sleep and/or circadian disruptions that could reasonably contribute to the functional impairment of post-ICU syndrome.

Our cohort of ARDS survivors reported moderately impaired sleep time and sleep efficiency on their sleep diaries, but these findings were less notable in the actigraphy data. Subjective sleep disturbance was pronounced, similar to prior questionnaire studies in ICU survivors (7). The prevalence of ISI-defined clinical insomnia at 3-months post-discharge was 28.6%, similar to the prevalence at 12-months post-discharge in our previous work of ICU survivors (26). Objective total sleep time is slightly better in our cohort compared than found among 1-month ICU survivors by Solverson et al. (TST_A_: 7.8 vs. 6.2 h, respectively); sleep efficiency estimates are quite similar (SE_A_: 81.9 vs. 78%, respectively) (41). The improved sleep time in our cohort compared to Solverson's may reflect methodological differences and/or the population studied (6, 7). We performed an average of 9 nights of actigraphy (Solverson et al. performed 3 nights). Given the potential for night-to-night variability, particularly among ICU survivors with insomnia (7, 42), a longer assessment period would be expected to generate more reliable sleep estimates. Factors that might explain slightly worse sleep in Solverson's cohort include the earlier assessment period (1 month post-ICU vs. 3 months after hospital discharge) and longer duration of mechanical ventilation (240 vs. 175 h, respectively). Solverson's study also included mostly medical ICU patients, who tend to be older and exhibit more pre-existing comorbidities than trauma ICU patients (57% of our cohort). Interestingly, our ARDS cohort exhibited higher severity of illness (APACHE II score: 24.5 vs. 20, respectively) and longer hospital length of stay (21 vs.15 days, respectively) than those in Solverson's cohort. The relative importance of pre-existing comorbidity and ICU factors in predicting post-ICU sleep disruption is a question that bears further investigation.

Most survivors in our cohort reported insomnia symptoms and subjective deficits in sleep quantity and quality, while standard actigraphy measures of sleep (total sleep time and sleep efficiency) were fairly normal. There are several possible reasons for these findings. First, sleep diary may align better with subjective sleep impairment than standard actigraphy measures, and sleep diary tends to underestimate sleep time and efficiency compared to actigraphy (22, 43, 44). Second, identifying circadian abnormalities requires specialized analyses that take into account the timing of the sleep period, which is not reflected in standard actigraphy parameters. Untreated circadian disruption may contribute to insomnia (45), and effective treatment must take account both sleep and circadian components. While limited in size and scope, this pilot study provides an intriguing potential insight into a circadian contribution to the subjective sleep complaints commonly described among survivors of critical illness (6, 42, 46).

### Rest-Activity Circadian Disruption in ARDS Survivors

Our cohort exhibited multiple circadian abnormalities including a less stable rest-activity circadian rhythm, later timings for their peak activity and rest periods, a greater variance in circadian phase, and less active and weaker rest-activity circadian strength compared to the community-dwelling US adult sample with similar age and BMI distribution. To our knowledge, the present study is the first using actigraphy to examine rest-activity circadian rhythms after critical illness.

Possible contributors to circadian disruption after hospitalization among ARDS survivors include inflammation, delirium, side effects of ICU treatments (i.e., sedative medications, mechanical ventilation), and post-ICU depression, anxiety, and post-traumatic stress disorder (3, 11–13, 47–49). Our cohort represented weaker rest-activity circadian rhythms compared to community-dwelling adults supported the lower values of cosinor magnitude as well as NPCRA amplitude and RA. A recent population-based study showed that lower relative amplitude of rest-activity rhythms was related to depressed mood, mood instability, and poor subjective wellbeing (i.e., happiness, health satisfaction) (50). A lack of regular daily routine may be another possible explanation for post-ICU circadian disruption, supported by findings in our cohort including the low IS value, large variance in sleep offset times, and high percentage of unemployed participants. Regular daytime routines (i.e., work or social schedules; exposure to light, exercise, and food cues) are essential to strengthen the regularity of the circadian system (9, 10, 51, 52). Further work is needed to identify post-ICU mental health and behavioral factors that may be associated with circadian disruption and serve as appropriate targets for intervention.

Left untreated, rest-activity circadian disruption may exacerbate cognitive and psychological comorbidities in ARDS survivors. Participants in our study were able to live independently and had mild functional limitations in activities of daily living, but IADLs only reflect some domains of cognitive function. ARDS survivors often experience persistent impairments in physical activity and social functioning (3, 4, 53), both of which have been found to be associated with low activity and weak circadian strength (54, 55). Impairments in memory, executive functioning, attention, visual-spatial construction, and language have been described in ARDS survivors but were not measured in this study (56, 57). In other populations rather than ICU survivors, cognitive impairment has been associated with disrupted rest-activity circadian rhythms (i.e., less stability, more fragmentation or lower rest-activity amplitude) (39, 55, 58–60). A prospective study with 1,282 healthy older community-dwelling women reported that a less stable, lower rest-activity amplitude, and delayed peak activity timing of rest-activity circadian rhythms predicted the development of either mild cognitive impairment or dementia 4.9 years later, independently of SE and TST (58). Future studies are needed to identify if rest-activity circadian disruption may be a contributor to long-term cognitive deficits among ARDS survivors.

Circadian re-entrainment may be a valuable treatment for sleep and quality of life improvement after critical illness (13, 36, 47). Re-entrainment generally includes a combination of chronotherapy (light and/or melatonin exposure) and behavioral interventions (e.g., set wake time). The timing of these therapies differs by the patient's underlying circadian phenotype. Delayed circadian phase (“night owl” phenotype) is treated with morning light and evening melatonin, while advanced circadian phase (“morning lark” phenotype) is treated with evening light. In our cohort, we discovered large variances in circadian phase markers of acrophase, M10, and L5 midpoints. Some subjects displayed delayed circadian phase (“night owl” phenotype) while others displayed advanced circadian phase (“morning lark” phenotype) (61). In the ICU, morning light and/or evening melatonin have been prescribed to improve nighttime sleep, circadian rhythms, and decrease delirium (13, 47). This timing of therapy is likely beneficial in patients with delayed circadian phase (e.g., “night owl” phenotype). However, it could worsen advanced circadian phase (e.g., “morning lark” phenotype) and may yield minimal benefit among those with normal circadian phase. Larger studies are needed, but our findings suggest that sleep interventions in ICU survivors may need to account for multiple circadian phenotypes.

### Limitations

Limitations of the current study include our small sample size and restriction of the sample to ARDS survivors. Our findings need replication in larger and more diverse samples of ICU survivors. Data analysis only included data from the ARDS survivors who completed actigraphy and sleep diary for 9 consecutive days. Participant bias may potentially limit our results to generalize a general population of ARDS survivors. Data on sleep prior to critical illness were not available, and therefore we cannot identify a causal role for critical illness in the circadian abnormalities found in our cohort. Our study suggests that actigraphy is a feasible method to identify circadian disruption in ARDS survivors. While actigraphy is a widely accepted method for estimating sleep and entrained circadian rhythms in other populations, its use has not formally been validated against PSG (the gold standard) in ARDS survivors. Additional study is warranted to examine the validity of actigraphy concurrently with PSG. Larger studies are needed to capture a more comprehensive picture of circadian rhythms after ARDS, potentially incorporating biological markers (i.e., melatonin) and measures of behavioral and environmental contributors (i.e., light exposure) that perpetuate circadian disruption.

## Conclusions

Findings from this pilot study show that sleep and rest-activity circadian disruption is common among survivors of acute respiratory failure 3 months after hospital discharge. Sleep and circadian disruption are potentially treatable through chronotherapy (i.e., light, melatonin) as well as behavioral strategies. Sleep improvement and circadian rhythm regularity may be a promising approach to improve quality of life and daytime function in ARDS survivors. Future research is warranted in larger samples to validate these findings, their association with quality of life, and to target specific chronotypes with effective circadian interventions.

## Data Availability Statement

The datasets generated for this study are available on request to the corresponding author.

## Ethics Statement

The studies involving human participants were reviewed and approved by the Subjects Institutional Review Board, University of Human Washington (No. 43516). The patients/participants provided their written informed consent to participate in this study.

## Author Contributions

EP contributed to design, patient enrollment, data collection, management, analysis, and interpretation. VK, SM, MV, and CH contributed to the design and implementation of the research. P-LY, TW, and RB performed data analysis and interpretation. P-LY, TW, RB, and EP took the lead in writing manuscript. All authors provided critical feedback to this manuscript and approved the final manuscript for publication.

### Conflict of Interest

The authors declare that the research was conducted in the absence of any commercial or financial relationships that could be construed as a potential conflict of interest.
